# Psychometric properties of the Opening Minds Stigma Scale for Health Care Providers in 32 European countries – A bifactor ESEM representation

**DOI:** 10.3389/fpubh.2023.1168929

**Published:** 2023-05-03

**Authors:** Dorottya Őri, Péter Szocsics, Tamás Molnár, Lucie Bankovska Motlova, Olga Kazakova, Sabrina Mörkl, Michael Wallies, Mohamed Abdulhakim, Sylvie Boivin, Krista Bruna, Carolina Cabacos, Elvira Anna Carbone, Elona Dashi, Giovanni Grech, Stjepan Greguras, Iva Ivanovic, Kaloyan Guevara, Selay Kakar, Konstantinos Kotsis, Ida Maria Ingeholm Klinkby, Jovana Maslak, Shevonne Matheiken, Ana Mirkovic, Nikita Nechepurenko, Angelis Panayi, Ana Telma Pereira, Edith Pomarol-Clotet, Shaeraine Raaj, Polona Rus Prelog, Joan Soler-Vidal, Robertas Strumila, Florian Schuster, Helena Kisand, Ann Hargi, Gumru Ahmadova, Matus Vircik, Helin Yilmaz Kafali, Natalia Grinko, Zsuzsa Győrffy, Sandor Rózsa

**Affiliations:** ^1^Institute of Behavioural Sciences, Semmelweis University, Budapest, Hungary; ^2^Department of Mental Health, Heim Pál National Pediatric Institute, Budapest, Hungary; ^3^Department of Psychology, Illinois Institute of Technology, Chicago, IL, United States; ^4^Department of Psychiatry and Psychotherapy, Semmelweis University, Budapest, Hungary; ^5^Department of Psychiatry, Aladar Petz County Teaching Hospital, Győr, Hungary; ^6^Division of Medical Psychology, 3rd Faculty of Medicine, Charles University, Prague, Czechia; ^7^Inpatient Psychiatric Department #2, Psychiatric Clinic of Minsk City, Minsk, Belarus; ^8^Division of Medical Psychology, Psychosomatics and Psychotherapy, Medical University of Graz, Graz, Austria; ^9^Psychiatric Hospital Littenheid, Sirnach, Switzerland; ^10^Department of Psychiatry, Vrije Universiteit Brussel, Brussels, Belgium; ^11^Department of Child and Adolescent Psychiatry, EPSM du Finistère Sud, Quimper, France; ^12^Admission Ward, State Psychiatric Hospital Gintermuiza, Jelgava, Latvia; ^13^Psychiatry Department, Centro Hospitalar e Universitário de Coimbra, Coimbra, Portugal; ^14^Department of Medical and Surgical Sciences, University Magna Græcia of Catanzaro, Catanzaro, Italy; ^15^Department of Neuroscience, University Hospital Center “Mother Theresa”, Tirana, Albania; ^16^Mental Health Services, Mount Carmel Hospital, Attard, Malta; ^17^Department of Psychiatry and Psychological Medicine, Division of Child and Adolescent Psychiatry and Psychotherapy, University Hospital Centre Zagreb, Zagreb, Croatia; ^18^Department for Child Psychiatry, Clinical Centre of Montenegro, Institute for Children’s Diseases, Podgorica, Montenegro; ^19^Acute Detoxification Ward, State Psychiatric Hospital for Treatment of Drug Addiction and Alcoholism, Sofia, Bulgaria; ^20^Department of Psychiatry, Erasmus University Medical Center, Rotterdam, Netherlands; ^21^Department of Psychiatry, University of Ioannina, Ioannina, Greece; ^22^Department of Child and Adolescent Psychiatry, Capital Region of Denmark, Copenhagen, Denmark; ^23^Institute of Mental Health, Belgrade, Serbia; ^24^Pennine Care NHS Foundation Trust, Oldham, United Kingdom; ^25^Child Psychiatry Unit, University Children's Hospital, University Medical Centre Ljubljana, Ljubljana, Slovenia; ^26^The Serbsky State Scientific Center for Social and Forensic Psychiatry, Moscow, Russia; ^27^Freelancer, Larnaca, Cyprus; ^28^Institute of Psychological Medicine, Faculty of Medicine, University of Coimbra, Coimbra, Portugal; ^29^Coimbra Institute for Biomedical Imaging and Translational Research, Coimbra, Portugal; ^30^FIDMAG Germanes Hospitalàries Research Foundation, Barcelona, Spain; ^31^Centro de Investigacion Biomedica en Red de Salud Mental, Instituto de Salud Carlos III, Barcelona, Spain; ^32^Department of General Adult Psychiatry, South Meath Mental Health Service, Meath, Ireland; ^33^Centre for Clinical Psychiatry, University Psychiatric Clinic Ljubljana, Ljubljana, Slovenia; ^34^Hospital Benito Menni, Complex Assistencial Salut Mental, Sant Boi de Llobregat, Spain; ^35^Department of Urgent and Post Urgent Psychiatry, CHU Montpellier, Montpellier, France; ^36^Institute of Functional Genomics, University of Montpellier, CNRS, INSERM, Montpellier, France; ^37^Psychiatric Clinic, Faculty of Medicine, Institute of Clinical Medicine, Vilnius University, Vilnius, Lithuania; ^38^Klinikum rechts der Isar der Technischen Universität München: Klinik und Poliklinik für Psychiatrie und Psychotherapie, Technische Universität München, Munich, Germany; ^39^University of Tartu, Tartu, Estonia; ^40^Department of Psychiatry, United City Hospital N15, Baku, Azerbaijan; ^41^Acute Psychiatric Department 1, Psychiatric Hospital Michalovce, Michalovce, Slovakia; ^42^Child and Adolescent Psychiatry, Sultanbeyli State Hospital, Istanbul, Türkiye; ^43^Department of Clinical Psychology, Ukrainian Catholic University, Lviv, Ukraine; ^44^Department of Personality and Health Psychology, Károli Gáspár University of the Reformed Church, Budapest, Hungary

**Keywords:** OMS-HC, stigma, mental health-related stigma, psychometrics, bifactor, bifactor ESEM, opening minds, psychiatrist

## Abstract

**Aims:**

To measure the stigma of healthcare providers toward people suffering from mental illness, the Opening Minds Stigma Scale for Health Care Providers (OMS-HC) is a commonly applied instrument. However, this scale has not been thoroughly validated in many European countries, its psychometric properties are still unknown and data on practicing psychiatrists is lacking. Therefore, this multicenter study aimed to assess the psychometric characteristics of the 15-item OMS-HC in trainees and specialists in adult and child psychiatry in 32 countries across Europe.

**Materials and methods:**

The OMS-HC was conducted as an anonymous online survey and sent *via* Email to European adult and child psychiatrists. Parallel analysis was used to estimate the number of OMS-HC dimensions. Separate for each country, the bifactor ESEM, a bifactor exploratory structural equation modeling approach, was applied to investigate the factor structure of the scale. Cross-cultural validation was done based on multigroup confirmatory factor analyses and reliability measures.

**Results:**

A total of 4,245 practitioners were included, 2,826 (67%) female, 1,389 (33%) male. The majority (66%) of participants were specialists, with 78% working in adult psychiatry. When country data were analyzed separately, the bifactor model (higher-order factor solution with a general factor and three specific factors) showed the best model fit (for the total sample χ^2^/df = 9.760, RMSEA = 0.045 (0.042–0.049), CFI = 0.981; TLI = 0.960, WRMR = 1.200). The average proportion of variance explained by the general factor was high (ECV = 0.682). This suggests that the aspects of ‘attitude,’ ‘disclosure and help-seeking,’ and ‘social distance’ could be treated as a single dimension of stigma. Among the specific factors, the ‘disclosure and help-seeking’ factor explained a considerable unique proportion of variance in the observed scores.

**Conclusion:**

This international study has led to cross-cultural analysis of the OMS-HC on a large sample of practicing psychiatrists. The bifactor structure displayed the best overall model fit in each country. Rather than using the subscales, we recommend the total score to quantify the overall stigmatizing attitudes. Further studies are required to strengthen our findings in countries where the proposed model was found to be weak.

## 1. Introduction

People with mental illness are frequently the targets of stigma and discrimination, which can have serious repercussions for both the stigmatized individuals and the larger society. Unfortunately, stigma affects people with mental illness not only in their daily lives but also when they seek medical care, including mental care, which results in poor physical and mental health of the person (1, 2). The experienced stigma from the service staff severely impacts the life expectancy and quality of life of individuals and serves as a predictor of their internalized stigma and disempowerment (3).

Developed to evaluate anti-stigma programs for healthcare workers, the Opening Minds Stigma Scale for Health Care Providers (OMS-HC) is one of the most widely used self-report measurements of stigmatizing attitudes worldwide. The authors of the scale created a pool of 50 items that they collected from existing scales and developed new items by consulting people who work in anti-stigma programs for healthcare providers. The pool was reviewed by professionals and people with lived experience, which was followed by cognitive interviewing of healthcare workers, and seven focus groups were held to improve the wording of the scale. Finally, the changes resulted in a reduction to 20 items (4). The factor analysis of the data resulted in a two-factor solution (attitude and disclosure) with 12 items that missed the important dimension of social distance. For this reason, the authors revisited the factor structure of the scale in a larger and more representative sample consisting of various healthcare providers that resulted in a more stable, three-factor solution (attitude, disclosure and help-seeking, and social distance) with 15 items (5). Studies with different samples of healthcare providers have shown OMS-HC scores to be a reliable and valid measure of stigmatizing attitudes toward patients in Italy (6), Hungary (7), Germany and Switzerland (8), Chile (9), and Singapore (10). The three dimensions of attitude, disclosure and help-seeking, and social distance structure appear to be similar; however, in most of the international studies, some items were found to be weak and loaded on different factors than that of the Canadian version and theory. Table 1 summarizes the factor analysis results on the 15-item OMS-HC in international studies.

**Table 1:** Overview of factor analyses results on the 15-item OMS-HC in international studies.

Research group	Investigated population	Method	Results		Country
			Structure	Model fit indices	
Modgill et al. (5)	Health care and social workers and medical students *n* = 1,305	PCA	3-dimensional structure	–	Canada
Destrebecq et al. (6)	Healthcare students *n* = 561	EFA	3-dimensional structure Item 15 has a poor factor loading on the Attitude factor	–	Italy
Chang et al. (10)	Nurse and medical students *n* = 1,002	ESEM	3-dimensional structure Item 1 was deleted Items 4, 5, 12 showed strong cross-loadings Items 5, 12 loaded on different factors	RMSEA = 0.069CFI = 0.948 TLI = 0.909	Singapore
Sapag et al. (9)	Primary healthcare workers *n* = 803	SEM	3-dimensional structure	RMSEA = 0.052 CFI = 0.832 TLI = 0.798	Chile
Őri et al. (11)	Child and adult psychiatrists *n* = 211	EFA, CFA	Higher order structure with a general factor and 3 specific factors Item 11 was reduced Items 13,14 showed crossloadings	RMSEA = 0.025 CFI = 0.961 TLI = 0.944	Hungary
Zuaboni et al. (8)	Staff in general psychiatric inpatient wards (*n* = 392)	EFA, CFA	3-dimensional structure Items 8, 11, 13 showed cross-loadings	RMSEA = 0.04 CFI = 0.92	Germany and Switzerland

Item numbers correspond to a 15-item scale.

PCA, principal component analysis; CFA, confirmatory factor analysis; EFA, exploratory factor analysis; ESEM, exploratory structural equation modeling (integration of EFA, CFA, and SEM); SEM, structural equation modeling; RMSEA, root mean square error of approximation; CFI, comparative fit index; TLI, Tucker-Lewis Index.

Participant samples of factor analytic studies included professionally heterogeneous samples, in which mental healthcare samples were composed predominantly of mental health nurses, medical students, and psychologists. Two psychometric studies on the scale included psychiatrists (subsample sizes in Germany and Switzerland together *n* = 49, Canada *n* = 79), and solely one study examined the factor structure of the scale on a psychiatrist sample (Hungary *n* = 211). Therefore, it is unknown whether the extent of findings could be generalized to the psychiatrist population, which is critical because investigating their stigmatizing attitudes toward people with mental health problems is considered an important and understudied area of research. Based on the current literature, the results are mixed; in some cases, their attitudes have been found to be more favorable, whereas in others they expressed more stigma than that of other healthcare providers (12–14). To ensure the cross-cultural applicability of the scale in evaluating the attitudes of psychiatrists, we aimed to choose a sample as homogeneous as possible; thus, the indicators of the instrument are not affected by characteristics of different occupations. The psychiatric community needs to have a measurement tested on a sample that consists solely of psychiatrists. It will allow using the OMS-HC in effectiveness and other measures of anti-stigma interventions among them, and the results can be interpreted in a cultural context. There is also a lack of multicenter studies in the literature on the attitudes of psychiatrists. However, to be able to perform cross-cultural measurements, the construct represented by the questionnaire needs to be simultaneously tested and confirmed by using different language versions.

Thus far, Chang in Singapore (10) and Sapag in Chile (9), have analyzed the psychometric properties of the OMS-HC by using exploratory structural equation modeling(ESEM). This method provides us with a suitable way to analyze attitude measurements because it combines the benefits of more sophisticated confirmatory factor analysis (such as goodness-of-fit or multigroup models) with less constrained exploratory factor analysis. Moreover, as opposed to confirmatory factor analysis, ESEM is expected to relieve more accurate correlations between latent factors, enhancing their discriminant validity, and leading to a more realistic representation of the data (15). The bifactor ESEM approach is a hierarchically ordered construct that provides a clear and explicit estimate of the overall stigmatizing attitude (general factor) along with specific factors that represent the individual features of each subscale that are not explained by the general factor (16). By allowing cross-loadings to be freely assessed among all elements used to reflect stigmatizing attitudes, this framework also considers the fallible nature of the indicators used to evaluate each construct (17).

No studies have scoped the factor structure of the OMS-HC through bifactor ESEM, and the OMS-HC has not yet been investigated across numerous psychiatrist samples from different countries. For these reasons, it is essential to know whether the OMS-HC scores measure the same construct in the same manner across psychiatrists from different countries with various cultural and ethnic backgrounds.

Based on the scientific literature, we aimed to test whether the three correlated factors or the bifactor ESEM approach to the OMS-HC is more appropriate in a diverse sample of practicing psychiatrists from 32 European countries. As an exploratory second aim, our study investigated the model-based reliability of the bifactor structure.

## 2. Materials and methods

### 2.1. Study design

In this cross-sectional research study, an anonymous online self-reported survey was distributed directly among adult and child psychiatrists in the participating 32 European countries. In each country, a dedicated psychiatrist investigator submitted the study protocol for review according to the local regulations, arranged the translation of the survey questions, and enrolled participants. In countries where the OMS-HC had not been available, it was translated according to the following guidelines: The English version was first translated into the local language by two different colleagues in psychiatry with a good command of English, then the two versions were reconciled into a single version, and then an independent translator back-translated it to English. An iterative procedure was used to resolve any discrepancies between the original and the back-translated versions of the scale; then, the final local version was sent to the participants. The survey was modified in case it was needed based on the comment of the first respondents. In countries with more than one official language, multilingual surveys (French, German, Italian in Switzerland, and Flemish and French in Belgium) were used to approach a broader population. Investigators distributed the questionnaire by contacting the national psychiatric associations, universities, local hospitals, and health centers and through professional networks, including social media platforms and professional groups’ email lists.

### 2.2. Participants

The combined total sample (*n* = 4,245) consisted of trainees and specialists in general adult and child psychiatry from 32 European countries. Not working in psychiatric care and medical student status were the exclusion criteria.

### 2.3. Measures

All participants completed the OMS-HC, and basic sociodemographic information (age range, gender, status, and place of work) was also gathered from them. The 15-item long OMS-HC is a self-report scale, which is conceptualized as an attitude measure comprising three components (attitude, disclosure and help-seeking, and social distance). Specifically, attitude is measured by six items (e.g., ‘*I struggle to feel compassion for a person with a mental illness*.’), disclosure and help-seeking, with four items (e.g., ‘*If I had a mental illness, I would tell my friends*.’), and social distance is measured by five items (e.g., ‘*I would not mind if a person with a mental illness lived next door to me*.’). Subjects indicate on a 5-point Likert scale the extent to which they identify themselves with the given statements from ‘strongly disagree’ to ‘strongly agree.’ The total scores range from 15 to 75, and the subscales from 6 to 30, 4 to 20, and 5 to 25, respectively. Higher scores reflect a more stigmatizing attitude. Before this study, the psychometric properties of the OMS-HC were investigated in Italy, Hungary, Germany, and Switzerland (German part) in the participating countries, and it was found to be a reliable and valid measurement of the stigmatizing attitudes (6–8).

### 2.4. Statistical analyses

The percentage (%) and sample size (n) are used to express the demographic data. First, using parallel analysis to compare the progressive eigenvalues from the given data matrix to those of a simulated data matrix created using random data of the same size, we determined the number of elements to be extracted (18). The Kaiser-Meyer-Olkin (KMO) measure of sampling adequacy was calculated to ensure that the matrices were suitable for exploratory factor (EFA) (should be >0.60). Bartlett’s Test of Sphericity was used to ensure the non-randomness of the correlation matrix (value of *p* should be 0.05). All confirmatory factor analyses and ESEM models used diagonally weighted least squares (WLSMV) to find the optimum model for each country. It accounts for standard errors and non-normality in chi-square test statistics for ordinal data. We tested unidimensional, three-factor, and bifactor model fit. We used the following indices to evaluate model fit: chi-square / degree of freedom (χ^2^/df) (<5.0), root mean square error of approximation (RMSEA, <0.06 as good fit, 0.06–0.08 as moderate fit, 0.08–0.1 as marginal fit, > 0.1 as poor fit), comparative fit index (CFI) and Tucker-Lewis Index (TLI), CFI and TLI (> 0.95 as good fit, >0.90 as acceptable, and < 0.90 as poor fit) (19). Model-based reliability was assessed by omega hierarchical (ωH), explained common variance (ECV), and percent uncontaminated correlations (PUC). These show that total and subscale scores accurately represent the target constructs. The ω (values range 0–1, with 0 indicating no reliability and 1 reflecting perfect reliability) can be used to estimate the factor’s contribution to summed (standardized) scores (20). Reise advised that the ωH should be greater than 0.50 and ideally greater than 0.75 (16). When PUC values are above 0.80, general ECV values predict bias less well. If the PUC values are less than 0.80, the general ECV values are larger than 0.60, and the ωH values are greater than 0.70, the instrument can be interpreted as unidimensional (16). Spearman’s correlation analyzed the associations between specific and general factors. IBM SPSS 26.0.0.0 (21) and MPlus Version 8.8 (22) statistical software were used for analysis.

## 3. Results

A total of *n* = 4,245 professionals in psychiatry completed the survey. The majority of the participants were female (*n* = 2,856, 67%), while young professionals between the ages of 24 and 35 years (*n* = 1856, 44%) were overrepresented in the sample. Most of them worked in adult psychiatry (*n* = 3,320, 78%) and inpatient facilities (*n* = 1884, 44%). Working facility of the participants and their years of experience in psychiatry are attached as a Supplementary material S1. There were no missing values in the dataset.

First, based on the suitable Bartlett’s Test of Sphericity (*p* < 0.0001) and the KMO measure of sampling adequacy (0.841) we performed an EFA on the pooled sample to see the factor matrix, the network of the items, and to create an overview of the structure of the scale. To determine the number of factors for extraction, parallel analysis was performed, and the eigenvalues were considered, which identified 3 or 4 different dimensions. As all item loadings were lower on the fourth factor than the other three, therefore, we continued with the three-factor structure that accounted for a sufficient amount of variance (25.1, 9.9, and 8.54%, respectively).

EFA results are found in the Supplementary material S2.

**Figure 1:**
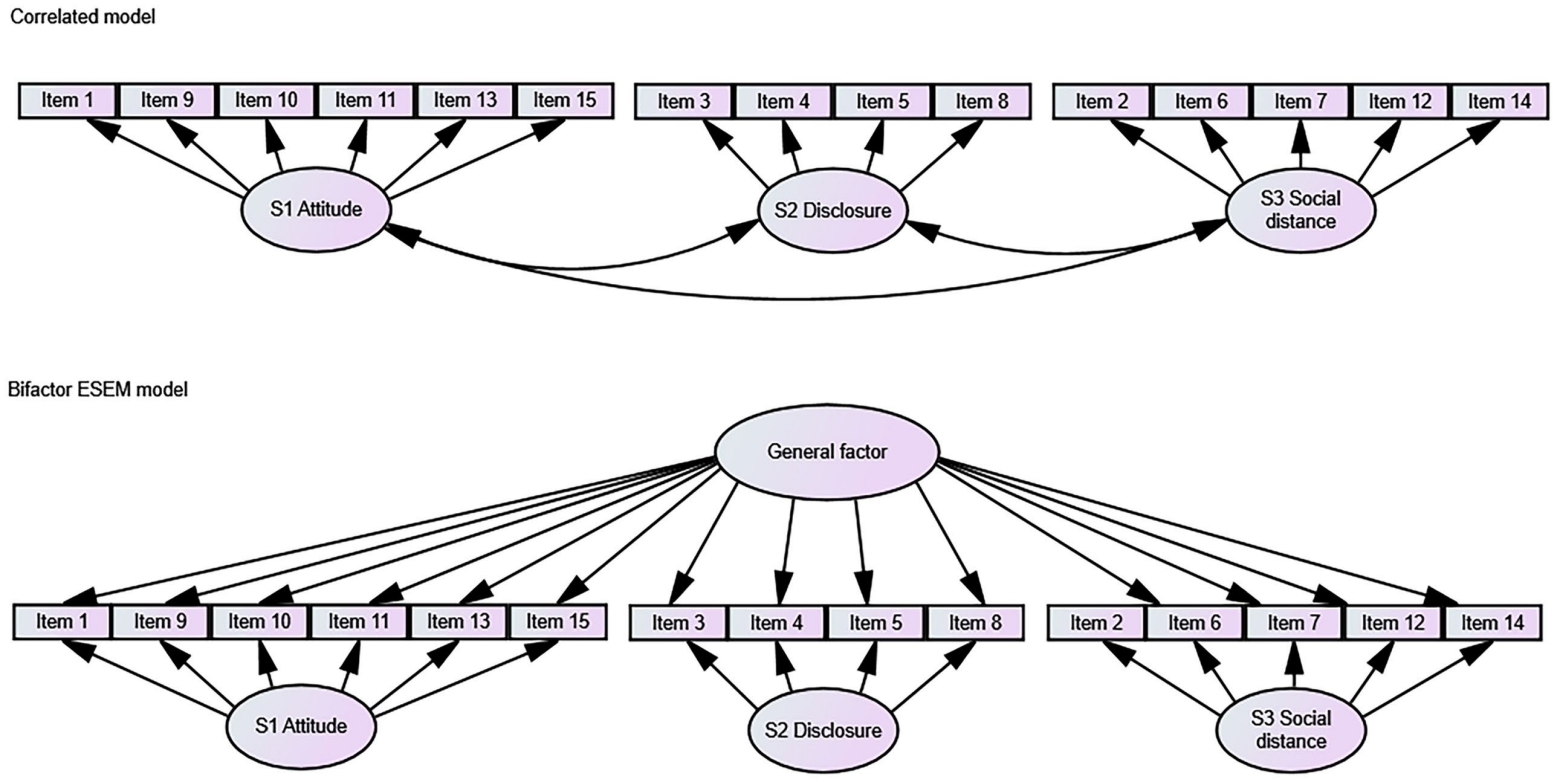
Simplified conceptual representations of the estimated models, ESEM cross-loadings (in the ESEM models all items are allowed to cross-load on all the specific-factors) are excluded in this figure to avoid cluttering.

A series of confirmatory factor analyses were performed to find the best model fit for the OMS-HC in each participating country. The bifactor ESEM model was found to be superior to the alternative models by showing an acceptable fitting level. This model (see Figure 1 for a comparison with the correlated model) consists of a general factor (loads directly onto all items) and three specific factors (load onto sub-groups of the same set of observed variables). The fit measures and the average loadings for the bifactor model are shown in Table 2. As presented, the model had excellent or acceptable levels of fit in most countries (29/32 countries). Across the 32 countries, the RMSEA ranged from 0.000 to 0.131 (*M* = 0.05, SD = 0.03), CFI ranged from 0.870 to 1.000 (*M* = 0.97, SD = 0.03), TLI ranged from 0.732 to 1,084 (*M* = 0.94, SD = 0.06), and the WRMR ranged from 0.304 to 0.651 (*M* = 0.47, SD = 0.07). However, the model failed to run in Albania and Swiss French and Swiss Italian languages due to the negative covariance matrices (theta). The absolute index, RMSEA, was moderate in Bulgaria, Cyprus, Greece, Italy, Latvia and Turkey, and it was associated with slightly lower than acceptable relative fit indices, CFI and/or TLI in Azerbaijan, Latvia, Montenegro, and Slovakia. The fit indices for the total sample were within the recommended ranges. Alternatively, the unidimensional and the correlated three-factor models were considered. The Supplementary material S3 contains their fit indices, which were poor and the models failed to run in several countries. In the unidimensional model, the RMSEA ranged from 0.078 to 0.172 (*M* = 0.12, SD = 0.02), the CFI ranged from 0.579 to 0.902 (*M* = 0.78, SD = 0.09), and TLI ranged from 0.509 to 0.886 (*M* = 0.75, SD = 0.10). The three-factor model showed a little better fit, with RMSEA ranging from 0.054 to 0.124 (*M* = 0.08, SD = 0.02), CFI and TLI ranging from 0.726 to 0.966 (*M* = 0.89, SD = 0.06) and 0.669 to 0.959 (*M* = 0.86, SD = 0.07), respectively. All fit indices indicate that the bifactor ESEM model provides us with the best model fit.

**Table 2:** Results of the bifactor ESEM model in each participating country.

Country	Language of the survey	Sample size	χ^2^	χ^2^/df	RMSEA	CFI	TLI	WRMR
Albania	Albanian	59	The residual covariance matrix is not positive definite					
Austria	German	133	64.722	1.269	0.045	0.985	0.968	0.452
Azerbaijan	Azerbaijani	35	81.633	1.600	0.131	0.870	0.732	0.511
Belarus	Russian	319	63.500	1.245	0.028	0.988	0.976	0.471
Belgium	Flemish	87	51.611	1.012	0.012	0.999	0.997	0.419
	Belgian French	19	45.026	0.883	0.000	1.000	1.084	0.333
Bulgaria	Bulgarian	65	69.882	1.370	0.075	0.955	0.908	0.456
Croatia	Croatian	87	66.885	1.311	0.060	0.975	0.949	0.452
Cyprus	Cypriot Greek	43	63.819	1.251	0.076	0.959	0.917	0.465
Czech Republic	Czech	222	76.890	1.508	0.048	0.974	0.946	0.491
Denmark	Danish	199	47.844	0.938	0.000	1.000	1.005	0.359
Estonia	Estonian	60	61.486	1.206	0.059	0.962	0.922	0.447
France	French	196	59.085	1.159	0.028	0.988	0.976	0.470
Germany	German	132	71.225	1.397	0.055	0.972	0.942	0.512
Greece	Greek	154	96.459	1.891	0.076	0.954	0.906	0.550
Hungary	Hungarian	211	73.013	1.432	0.045	0.967	0.933	0.508
Ireland	English	75	48.208	0.945	0.000	1.000	1.004	0.304
Italy	Italian	170	104.037	2.039	0.078	0.981	0.960	0.530
Latvia	Latvian	101	83.278	1.632	0.079	0.937	0.870	0.508
Lithuania	Lithuanian	77	54.547	1.069	0.030	0.988	0.976	0.438
Malta	English	44	62.698	1.229	0.072	0.971	0.941	0.437
Montenegro	Montenegrin	35	60.958	1.195	0.075	0.928	0.852	0.478
Netherlands	Dutch	170	79.483	1.588	0.057	0.968	0.935	0.519
Portugal	Portugal	148	66.298	1.299	0.045	0.986	0.971	0.444
Russia	Russian	206	66.336	1.301	0.038	0.986	0.970	0.440
Serbia	Serbian	52	53.300	1.045	0.029	0.994	0.988	0.405
Slovakia	Slovak	77	83.061	1.629	0.090	0.896	0.786	0.559
Slovenia	Slovenian	90	67.360	1.321	0.060	0.970	0.939	0.467
Spain	Spanish	159	68.763	1.348	0.047	0.970	0.938	0.476
Switzerland	Swiss German	365	129.558	2.540	0.065	0.961	0.920	0.651
	Swiss French	75	The residual covariance matrix is not positive definite					
	Swiss Italian	13	The sample correlation of item 7 and item 13 is −1					
Turkey	Turkish	146	93.852	1.840	0.076	0.969	0.937	0.562
Ukraine	Ukrainian	52	61.205	1.200	0.062	0.952	0.902	0.476
United Kingdom	English	169	70.228	1.377	0.047	0.988	0.975	0.417
Total sample	–	4,245	497.784	9.760	0.045	0.981	0.960	1.200

χ^2^, chi-square; df, degree of freedom; RMSEA, root mean square error of approximation; CFI, comparative fit index; TLI, Tucker-Lewis Index; WRMR, weighted root mean square.

The specific and general factor of the OMS-HC were strongly correlated: Attitude: *r* = 0.791, *p* < 0.0001; Disclosure: *r* = 0.680, *p* < 0.0001; Social distance: *r* = 0.785. Additionally, the specific factors correlated statistically significantly with each other: Attitude and Disclosure: *r* = 0.294, *p* < 0.001; Attitude and Social distance: *r* = 0.506, *p* < 0.0001; Disclosure and Social distance: *r* = 0.310, *p* < 0.0001.

In the next step, we tested the model-based reliability (see Table 3). The general factor indicated acceptable model-based reliability for the whole sample. However, the reliability of the specific factors was found to be poor. Among the specific factors, the Disclosure and Help-seeking was the one that approached the limit of acceptable reliability. As the PUC value for the model was 0.705 and ECV and ωH of the general factor were 0.682 and 0.746, respectively.

**Table 3:** Estimates of model-based reliability of the general and specific factors.

	ECV	ω	ωH	PUC
General factor	0.682	0.854	0.746	0.705
Specific factor 1 Attitude	0.119	0.731	0.227	
Specific factor 2 Disclosure and help-seeking	0.179	0.631	0.481	
Specific factor 3 Social distance	0.020	0.769	0.007	

ECV, explained common variance; ω, coefficient omega; ωH, coefficient omega hierarchical; PUC, percent of uncontaminated correlations.

## 4. Discussion

The primary aim of this study was to examine the factor structure of the OMS-HC in 32 countries across Europe on a large group of psychiatrists. To achieve our goal, after gaining an insight into the dimensionality of the pooled sample by parallel analysis, we performed a CFA series and compared the fit indices of the unidimensional, the three-correlated, and the bifactor ESEM models for each participating country. We specifically examined whether the proposed bifactor ESEM model of the OMS-HC is replicable across cultures and whether it more accurately captures the factor structure in comparison to competing models, especially the three-factor model proposed by Modgill (5), which was replicated in Italy, Chile, Singapore and on a German-Swiss sample (6, 8–10).

Both the parallel analysis and the EFA, strongly supported the 3-dimensionality of the scale on the pooled sample. Therefore, it has been our starting point for investigating the structure. The simple three-dimensional structure would have been in line with the Canadian, Italian, Chilean, and German-Swiss results. However, note that these studies detected significant cross-loadings of items between factors. Moreover, one item was removed from the analyses in Singapore due to poor loading across all three factors.

Because of the inherent limitations of the simple three-factor structure, we decided to investigate the model further. Our research has demonstrated that the bifactor model provided us with the best approximation of the factor structure of the OMS-HC with a good to acceptable fit in nearly all countries. It seemed superior to alternative models, including the three-factor correlated trait model based on the fit indices. In fact, our model does not contradict the three-factor model; furthermore, it investigates it for a higher-order solution. The three-dimensions are identified as three specific factors, supplemented by a general factor in a hierarchical structure resulting in better model fit. Although it showed a good fit in most countries, the final model failed to run in Albania and on the Swiss-French and Swiss Italian samples due to negative covariance matrices. Despite having a model that fits perfectly by definition, this error message is not unexpected with small sample sizes, in which simulation methods could cause more sampling errors (23). Moreover, the bifactor model is relatively complex and contains many parameters, which could be even more complicated with small samples. Accordingly, this could not have been prevented in countries where the total number of psychiatrists is low or the investigator had difficulties enrolling a larger sample. In Albania, the enrollment rate was 71% (*n* = 59 were enrolled from the total of *n* = 83 psychiatrists), which is considered a very high completion rate - especially among psychiatrists (24). In Switzerland, quite a large number of practitioners work in psychiatry (*n* = 3,848); however, we had no information about the exact number of French or Italian-speaking psychiatrists. Participants could choose the language they were most comfortable with in countries with multiple official languages. Both the Swiss French and Swiss Italian samples were quite small (*n* = 75 and *n* = 13, respectively), which exposed them to this type of error. The small sample size was probably the reason behind the Montenegrin and Azeri lower fit indices as well. Consequently, in these countries, thorough exploratory factor analysis is recommended to understand the psychometric properties of the scale. Its investigation on larger and more diverse samples (for example, with the involvement of different mental healthcare workers: e.g., psychologists, nurses, and other medical staff) could also be beneficial, particularly in those countries where the model-fit was poor.

We could not identify specific items with serious cross-loadings that were problematic in most samples. Such as item 1(‘*I am more comfortable helping a person who has a physical illness than I am helping a person who has a mental illness*.’) found to be weak in Singaporean medical and nursing students (10) or item 8 (‘*If I had a mental illness, I would tell my friends*.’) and 13 (‘*Health care providers do not need to be advocates for people with mental illness*.’) loaded on different factors than expected in German and Swiss healthcare workers on psychiatric wards (8). Item 11 (‘*More than half of people with mental illness do not try hard enough to get better*.’) was removed from the scale in a Hungarian study on psychiatrists due to poor loading across all three factors (7). In this study, different items were found to be weak in countries where the fit indices were lower than the acceptable range. Therefore, item reduction and corresponding abbreviation of the scale were not an option for finding a better fitting solution for each country.

The model-based reliability results suggest using the general factor rather than the specific factors (16). Based on our results, the disclosure and help-seeking factor were the most reliable among the specific factors, whereas this and the general factor explained a substantial proportion of the variance. These findings indicate that using the total score to quantify the overall stigmatizing attitudes and the disclosure and help-seeking subscale to gain information about the help-seeking behavior may be the best choices when we seek reliable stigma measurements, and we would like to make a comparison between countries.

The correlations among the specific factors were weak but statistically significant, supporting the notion of a shared conceptual theme. To note, the correlation coefficients in this study were lower than those in Canada (5).

In summary, this is the first study to examine the factor structure of the OMS-HC in a large sample of psychiatrists across 32 European countries by using the bifactor ESEM approach. We believe the reported findings are significant from a theoretical and an applied point of view. From a theoretical perspective, a bifactor model enables the investigation of the degree to which specific factors are independent of the general factor; thus, it may associate differently with other mental health determinants and outcomes. In addition, our model provides an upgraded version of Modgill’s three-factor model; at the same time, the bifactor approach also supports the validity of their model as comprised of three related components (5). Regarding the applied perspective, it is important to gain a better understanding of whether the OMS-HC could be used as an appropriate and reliable attitude measurement for psychiatrists. Moreover, the analyses will also help broaden the understanding of cross-cultural differences because it is important to recommend conducting psychometric analyses before evaluating the possibility of generalizing findings in different cultural contexts. However, we recommend doing this with caution, and future research must investigate the psychometric properties of the scale in countries where the model failed to run or the fit indices were not within the acceptable ranges. Finally, having a valid and reliable measure for provider stigma toward people with mental health problems in several European countries will allow us to measure and tailor anti-stigma programs for mental healthcare workers. Also, the findings may help guide future studies to evaluate and put into context the factor structure of the OMS-HC in other countries.

The findings in the study must be viewed with some limitations in mind. Firstly, we aimed to approach all practicing psychiatrists in each country; therefore, national psychiatric associations were also asked to disseminate the survey. However, despite all efforts, it was not possible to obtain nationally representative samples. Therefore, the convenience sampling approach is a possible limitation of our study. Secondly, in our research, young colleagues were overrepresented (44% of the subjects were 24–35 years of age); they were probably easier to approach with online questionnaires. Thirdly, this study examined a sample consisting solely of psychiatrists. It is uncertain if the findings apply to other healthcare workers, not only for mental healthcare providers. Lastly, most of the data were collected during the COVID-19 period. We have no data on how their attitudes would be without the challenges brought on by the pandemic. It is not yet known whether the pandemic has or not a significant effect on their stigmatizing attitudes; however, the overwhelming workload and the related stress might bring them more challenges than before (11).

## 5. Conclusion

Our results agree with the three-dimensional structure of the OMS-HC proposed by the scale authors after the modification and abbreviation of the scale to 15 items. In addition, however, we upgraded the investigation of the scale from a first-order factor solution to a bifactor ESEM model, which seemed more appropriate and provided us with better fit indices in a large sample of psychiatrists from several European countries. The model fit was good or acceptable in most nations; however, based on the model-based reliability results, the general factor and the disclosure and help-seeking specific factor explained substantial variance. Consequently, we recommend using the total score of the OMS-HC and probably the disclosure and help-seeking subscale scores rather than other subscales when we would like to measure the stigma in the participating countries.

## Data availability statement

The raw data supporting the conclusions of this article will be made available by the authors, without undue reservation.

## Ethics statement

The study that involved human participants was conducted in accordance with the principles of the Declaration of Helsinki. Participants provided electronic informed consent prior to completing the online survey (by clicking `yes’). The Hungarian core study was approved by the Regional and Institutional Committee of Science and Research Ethics of the Semmelweis University, Budapest, Hungary (SE-RKEB: 189/2019), followed by site approvals in participating countries. The ethics revision process for online survey projects was required in the following countries based on the written statement of the local investigators, and the study was approved by the following ethics committees. Albania: Albanian Medical Ethics Committee (Nr. 303/13). Austria: Ethics Committee of the Medical University of Graz (32-619 ex 19/20). Belarus: Ethical Committee of the Belarusian Psychiatric Association (1/2020), Belgium: Ethics committee of the University Hospital Brussels (2021/011), Croatia: Ethics Committee of the University Hospital Center Zagreb (8.1.-21/120-2, 02/21 JG), Cyprus: Cyprus National Bioethics Committee (2020.01.172.), Czech Republic: The Ethical Committee of the Third Medical Faculty, Charles University (11/2020), Estonia: Tartu University Ethics Committee, Tartu, Estonia (322/T-9), Germany: Ethikkommission der Technischen Universität München (679/20S), Greece: University of Ioannina (2638/16-7-2020), Ireland: Royal College of Physicians of Ireland Research Ethics Committee (RCPI RECSAF 134), Malta: Health Ethics Committee (HEC13/2020) Netherlands: Medical Ethics Review Committee of Erasmus Medical Center (MEC-2021-0151), Portugal: Ethical Committee of the Faculty of Medicine, University of Coimbra (CE-136/2020), Serbia: Ethics Committee of the Institute of Mental Health, Belgrade (1060/2094/1), Turkey: Research Ethics Committee of the Ministry of Health Ankara City Hospital (E1/928/2020), and United Kingdom: Pennine Care NHS Foundation.

## Author contributions

DŐ was the principal investigator of the study and the major contributor to the study design, writing of the manuscript, and data analysis. PS and TM as members of the Hungarian core team, conceived and planned the study and helped in writing the manuscript. SRó being a professional statistician, verified the analytical methods, suggested and developed codes for the BESEM, helped to interpret the results, improved the manuscript with his expert comments, and also supervised the project with ZG. LB, OK, SMö, MW, MA, SB, KB, CC, EC, ED, GG, SG, II, KG, SK, KK, IK, JM, SMa, AM, NN, AP, ATP, EP-C, SRa, PR, JS-V, RS, FS, HK, AH, GA, MV, HY, and NG were essential contributors, planned the local data collection in each of their countries, translated the questionnaire to their local language(s), provided active assistance in the data collection and contributed to the manuscript writing. All authors have read and approved the final version of the manuscript and given their consent for publication.

## Funding

DŐ was awarded the National Youth Talent Award in 2020 and 2021 (Ministry of Human Resources, Hungary, (NTP-NFTÖ-20-B-0134 and NTP-NFTÖ-21-B-0280) and the Prominence Award of the Kerpel-Fronius Talent Support Program of Semmelweis University in 2021 (EFOP-3.6.3-VEKOP-16-2017-00009), which covered the expenses of the 1-year account of the online survey platform and the dissemination of the study results at international congresses. SRó was supported by the Károli Gáspár University of the Reformed Church (Grant No. 20754B800).

## Conflict of interest

The authors declare that the research was conducted in the absence of any commercial or financial relationships that could be construed as a potential conflict of interest.

## Publisher’s note

All claims expressed in this article are solely those of the authors and do not necessarily represent those of their affiliated organizations, or those of the publisher, the editors and the reviewers. Any product that may be evaluated in this article, or claim that may be made by its manufacturer, is not guaranteed or endorsed by the publisher.

## Abbreviations

CFI: comparative fit index
df: degree of freedom
ECV: the explained common variance
EFA: exploratory factor analysis
ESEM: exploratory structural equation modeling
KMO: Kaiser-Meyer-Olkin
OMS-HC: Opening Minds Stigma Scale for Health Care Providers
PCA: Principal component analysis
PUC: percent of uncontaminated correlations
RMSEA: root mean square error of approximation
SEM: structural equation modeling
TLI: Tucker-Lewis Index
WLSMV: diagonally weighted least squares
WRMR: Weighted Root Mean Square Residual
χ^2^: chi-square
ωH: coefficient omega hierarchical
