# Supplementary material for: Psychometric properties of the Opening Minds Stigma Scale for Health Care Providers in 32 European countries – A bifactor ESEM representation
Source: Front Public Health. 2023 May 3;11:1168929. doi: 10.3389/fpubh.2023.1168929 (PMC10285467; doi:10.3389/fpubh.2023.1168929)
Supplement: Supplementary file 1 [file Table_1.pdf]

S1. Years of experience in psychiatry of the participants and their working facilities

Country	Years of experience in psychiatry						Working facility						
	0-5	6-10	11-20	21-30	31-40	>40	inpatient hospital	outpatient service	other outpat where psych patients are treated	daycare service	exclusively private practice	do not work in patient care	other
Albania	38	5	10	5	1	0	44	9	0	0	4	0	2
Austria	36	41	29	19	6	2	93	6	1	0	26	1	6
Azerbaijan	23	8	4	0	0	0	13	15	1	1	1	3	1
Belarus	131	70	81	31	6	0	104	139	24	11	11	14	16
Belgium	48	23	25	7	3	0	60	27	4	4	9	0	2
Bulgaria	21	9	6	19	9	1	43	13	0	3	6	0	0
Croatia	31	31	11	11	3	0	43	37	0	6	0	0	1
Cyprus	10	11	16	4	2	0	13	8	2	0	18	0	2
Czech Republic	47	39	28	63	27	18	119	67	4	2	26	0	4
Denmark	70	50	40	25	13	1	73	104	4	1	5	8	4
Estonia	21	10	11	3	9	6	35	17	2	0	5	0	1
France	61	39	58	21	14	3	78	60	35	6	9	1	7

Germany	35	27	35	27	5	3	80	32	2	8	8	2	0
Greece	26	35	52	28	10	3	27	56	11	1	53	1	5
Hungary	84	49	30	27	11	9	139	55	2	5	9	1	0
Ireland	23	36	10	5	1	0	31	28	9	2	0	0	5
Italy	65	29	30	28	16	2	74	61	7	10	13	2	3
Latvia	37	15	9	16	15	9	67	15	9	2	7	0	1
Lithuania	19	18	17	16	6	1	37	26	2	3	7	0	2
Malta	17	9	11	3	3	1	21	20	1	0	2	0	0
Montenegro	10	8	10	6	1	0	23	10	0	1	0	0	1
Netherlands	35	27	48	34	23	3	36	87	3	1	19	0	24
Portugal	71	40	15	6	15	1	57	75	2	1	4	1	8
Russia	130	28	24	16	7	1	113	36	14	5	21	5	12
Serbia	30	12	8	2	0	0	30	6	1	12	2	1	0
Slovakia	19	21	14	12	4	7	48	22	1	0	4	0	2
Slovenia	41	17	14	11	6	1	63	24	1	2	0	0	0
Spain	28	28	33	41	25	4	54	70	4	10	7	2	12
Switzerland	158	85	70	82	46	12	154	112	19	8	150	3	7
Turkey	30	78	29	3	5	1	11	123	3	0	6	2	1
Ukraine	17	10	19	5	1	0	29	14	1	0	5	2	1
United Kingdom	55	43	49	19	3	0	72	82	7	0	0	0	8
