# Supplementary material for: Psychometric properties of the Opening Minds Stigma Scale for Health Care Providers in 32 European countries – A bifactor ESEM representation
Source: Front Public Health. 2023 May 3;11:1168929. doi: 10.3389/fpubh.2023.1168929 (PMC10285467; doi:10.3389/fpubh.2023.1168929)
Supplement: Supplementary file 2 [file Table_2.pdf]

S2. The factor structure of the 15-item version of the OMS-HC

Items	Original subscale	Factors		
		1 (Social distance	2 (Disclosure)	3 (Attitude)
2 ‘willing to live with’	Social distance	**0.41**		
6 ‘hire’	Social distance	**0.54**		
7 ‘physicians’	Social distance	**0.70**		
12 ‘children’	Social distance	**0.51**		
14 ‘next-door’	Social distance	**0.38**		
3 ‘disclose’	Disclosure		**0.62**	
4 ‘weak’	Disclosure		**0.50**	
5 ‘reluctant’	Disclosure		**0.48**	
8 ‘telling friends’	Disclosure	0.39	**0.45**	
1 ‘comfort’	Attitude			**0.43**
9 ‘react’	Attitude			**0.44**
10 ‘little’	Attitude			**0.57**
11 ‘try’	Attitude			**0.30**
13 ‘advocate’	Attitude			**0.30**
15 ‘compassion’	Attitude			**0.41**

The principal axis factoring method was used with direct oblimin rotation. For the sake of clarity, factor loadings lower than 0.3 are hidden, and the highest factor loadings are highlighted in bold.
