# Supplementary material for: Psychometric properties of the Opening Minds Stigma Scale for Health Care Providers in 32 European countries – A bifactor ESEM representation
Source: Front Public Health. 2023 May 3;11:1168929. doi: 10.3389/fpubh.2023.1168929 (PMC10285467; doi:10.3389/fpubh.2023.1168929)
Supplement: Supplementary file 3 [file Table_3.pdf]

S3. Fit indices for the unidimensional and the three-factor models

Country	Unidimensional model					Three-correlated model				
	χ^2	df	RMSEA	CFI	TLI	χ^2	df	RMSEA	CFI	TLI
Albania	162.012	90	0.116	0.833	0.805	140.755	87	0.102	0.875	0.849
Austria	197.940	90	0.095	0.879	0.859	149.871	87	0.074	0.929	0.915
Azerbaijan	168.127	90	0.157	0.669	0.613	The model is not identified				
Belarus	262.610	90	0.078	0.840	0.813	169.122	87	0.054	0.924	0.908
Belgium (Flemish)	161.564	90	0.096	0.833	0.805	135.870	87	0.080	0.886	0.862
Belgium (French)	106.091	90	0.097	0.890	0.872	The model is not identified				
Bulgaria	262.960	90	0.172	0.589	0.520	The model is not identified				
Croatia	167.950	90	0.100	0.879	0.858	149.200	87	0.091	0.903	0.883
Cyprus	140.439	90	0.114	0.841	0.814	139.208	87	0.118	0.835	0.801
Czech Republic	281.136	90	0.098	0.806	0.774	174.265	87	0.067	0.912	0.893
Denmark	321.851	90	0.114	0.846	0.820	153.998	87	0.062	0.955	0.946
Estonia	186.656	90	0.134	0.651	0.593	134.064	87	0.095	0.830	0.795
Flemish	161.564	90	0.096	0.833	0.805	135.870	87	0.080	0.886	0.862
France	241.714	90	0.093	0.783	0.747	139.074	87	0.055	0.925	0.910
Germany	192.799	90	0.093	0.857	0.834	139.193	87	0.067	0.928	0.913
Greece	309.430	90	0.126	0.780	0.743	The model is not identified				
Hungary	266.606	90	0.096	0.738	0.695	169.886	87	0.067	0.877	0.852
Ireland	244.766	90	0.151	0.896	0.879	The model is not identified				
Italy	357.225	90	0.132	0.902	0.886	314.449	87	0.124	0.917	0.900
Latvia	240.487	90	0.129	0.705	0.656	211.327	87	0.119	0.756	0.706
Lithuania	156.015	90	0.098	0.784	0.748	121.438	87	0.072	0.887	0.864
Malta	183.076	90	0.153	0.770	0.732	The model is not identified				

Montenegro	144.358	90	0.131	0.609	0.544	The model is not identified				
Netherlands	248.593	90	0.102	0.824	0.795	168.721	87	0.074	0.910	0.891
Portugal	313.200	90	0.129	0.797	0.763	The model is not identified				
Russia	345.390	90	0.117	0.761	0.721	241.744	87	0.093	0.855	0.825
Serbia	176.776	90	0.136	0.784	0.748	The model is not identified.				
Slovakia	179.176	90	0.113	0.710	0.662	138.383	87	0.088	0.833	0.799
Slovenia	260.088	90	0.145	0.692	0.640	The model is not identified				
Spain	230.106	90	0.099	0.762	0.722	177.501	87	0.081	0.846	0.815
Swiss French	159.787	90	0.102	0.894	0.877	109.310	87	0.058	0.966	0.959
Swiss German	521.856	90	0.115	0.785	0.749	282.152	87	0.078	0.903	0.883
Swiss Italian	The weight matrix is not positive definite					The weight matrix is not positive definite				
Turkey	271.827	90	0.118	0.869	0.848	180.894	87	0.086	0.933	0.933
Ukraine	180.156	90	0.139	0.579	0.509	145.715	87	0.114	0.726	0.669
United Kingdom	427.335	90	0.149	0.785	0.749	156.306	87	0.069	0.956	0.947
